# Air Pollution Reduces Interpersonal Trust: The Roles of Emotion and Emotional Susceptibility

**DOI:** 10.3390/ijerph18115631

**Published:** 2021-05-25

**Authors:** Yubo Hou, Meiqi Gao, Lianqiong Huang, Qi Wang

**Affiliations:** 1 School of Psychological and Cognitive Sciences and Beijing Key Laboratory of Behavior and Mental Health, Peking University, Beijing 100871, China; gmq0520@gmail.com (M.G.); h_lianqiong1992@163.com (L.H.); 2 Department of Human Development, Cornell University, Ithaca, NY 14853, USA

**Keywords:** air pollution, haze, interpersonal trust, emotion, emotional susceptibility

## Abstract

Air pollution has been shown to have detrimental effects on physical and mental health, yet little is known about how air pollution affects psychosocial functioning in everyday life. We conducted three studies that utilized experimental methods and web crawler technology to examine the effect of hazy environmental conditions on perceived interpersonal trust, and to investigate the roles of emotion and emotional susceptibility in mediating or moderating the negative impact of air pollution. In Study 1, participants were presented with landscape photos that showed either hazy scenes or clear scenes. Those who viewed photos of hazy scenes reduced their levels of interpersonal trust. In Study 2, emotion data were collected from social media with web crawler technology, in connection with meteorological monitoring data during the same period. Hazy conditions were associated with reduced expressions of positive emotion on social media, whereas clearer conditions were associated with enhanced positive emotional expressions. In Study 3, we simulated Weibo communications in the laboratory. The findings showed that emotional susceptibility moderated the negative effect of hazy conditions on interpersonal trust, and negative emotion mediated the effect of hazy conditions on interpersonal trust. The findings advance the understanding of the impact of air pollution on interpersonal trust and social relations and the associated psychological mechanisms and boundary conditions. They have important real-life implications.

## 1. Introduction

Air pollution strongly affects the quality of human life. It deprives people of the pleasure of viewing blue skies and natural scenery, eradicates the joy of travelling to scenic attractions, increases anxiety about health, and in worst cases, causes illness and death. Indeed, air pollution and the diseases it causes resulted in the death of more than five million people in 2017, according to the U.S. data reported in 2019 (https://www.healtheffects.org; accessed on 12 February 2019). Air pollution is now the leading cause of death in India. Britain regards air pollution as one of the greatest challenges to public health. China considers the reduction of haze caused by air pollution to be its top priority. It is therefore important to further understand the negative impact of air pollution on human experience and behavior.

Extensive research has revealed the detrimental effects of air pollution on physical health, and the findings have served as the basis for preventions, interventions, and policy making [1,2]. Some research has also been done in the field of psychology to understand abnormal psychological states and mental diseases caused by air pollution [3,4]. Yet little is known about the effects of air pollution on psychosocial functioning among healthy individuals in everyday life, which can have long-term consequences for work productivity, health, and social cohesion. To fill the gap, the present research examines the impact of air pollution on interpersonal trust and the associated psychological mechanisms and boundary conditions.

### 1.1. Air Pollution and Its Negative Consequences

Mental and public health experts have observed a link between meteorological environmental data and psychological states. In one study, for example, researchers collected daily data of psychiatric emergency room visits for two consecutive summers and obtained corresponding weather and pollutant data from the National Oceanic and Atmospheric Administration (NOAA) database [5]. They found that low barometric pressure and high cloud cover were positively associated with the number of patients admitted to emergency rooms due to depression, as well as the number of schizophrenia cases. After controlling for social factors such as unemployment in the data from 1991 to 2008 on suicides and air pollution, researchers found that sulfur dioxide, ozone, and other pollutants were associated with long-term suicide rates [6]. The various pollutants are also damaging for physical health: Aerosols cause acute respiratory problems, while gases penetrate deeply into human organs and cause long-term neural and metabolic deficiencies. An observational cohort study showed a significant correlation between PM_2.5_ and anxiety symptoms among elderly women (*M*_age_ = 70) [7]. Air pollution is further linked to reduced prosocial tendencies by inducing negative attitudes and anxiety toward the outside world and strangers, reducing interpersonal attraction, and increasing the likelihood of immoral behaviors [8]. An analysis of crime rates in multiple cities in the U.S. over the past nine years showed that air pollution predicted the rate of six major crimes [9].

Laboratory research has also examined the psychological impact of air pollution. One study found that when participants were isolated in an environment permeated with unpleasant odors, they showed increased negative feelings and attitudes toward others; yet when the participants shared their environment-elicited stress with others, they had increased interpersonal attraction and increased willingness to cope together [8]. Air pollution can also be manipulated on psychological levels: When participants viewed pictures of hazy weather and imagined living in those conditions, they showed increased unethical behaviors [9]. Notably, it is difficult in laboratory studies to artificially produce small-scale air pollution just by releasing odiferous gases to simulate polluted air. To avoid artificial intervention and to make research more ecologically valid, studies need to be conducted in actual weather conditions. In one study, for example, researchers in India visited two cities with significant climate differences and investigated emotions and helping behaviors in people who were visiting the local zoos [10]. They found that people were more cheerful and more willing to help others when the weather was warm. This study, however, focused on the effect of weather rather than air pollution.

Network technology and precise positioning technology offer additional ways to study the effects of air pollution. For example, Roberts et al. [11] collected data on depression, anxiety, and behavioral disorders of twins at each stage of growth from 5 to 18 years old, and estimated air pollution data such as NO and PM_2.5_ based on the latitude and longitude of their residences. The researchers found that exposure to air pollutants was not associated with depressive symptoms or behavioral disorders before age 12, but it was by age 18. Previous studies have primarily focused on the effects of air pollution on physical and mental health. Air pollution may also influence psychosocial functioning such as interpersonal trust.

### 1.2. Interpersonal Trust and the Contributing Factors

Interpersonal trust is the generalized expectancy that others can be relied upon [12]. It occurs when individuals feel safe about relying on interpersonal communications and is essential for maintaining good interpersonal relationships [13,14]. Research has shown that interpersonal trust reduces management costs, improves organizational effectiveness, stimulates cooperation and altruism among employees, and further helps organizations achieve their goals [15,16,17]. When employees trust their leaders, they are more willing to follow instructions, rules, and regulations and accept the organization’s conflict resolution proposals [18]. Without interpersonal trust, people tend to focus on fraud prevention and transaction security, which eventually increases transaction costs [19]. At the national level, interpersonal trust is related to life satisfaction among citizens [20].

Individuals tend to trust others with positive traits such as integrity, consistency, openness, professionalism, interpersonal skills, benevolence, and self-control [21,22]. Studies have further shown that interpersonal trust is influenced by a variety of family, societal, and cultural factors [12,23]. For example, a study of 29 countries and regions found that citizens from countries with higher incomes and lower levels of inequality had higher levels of interpersonal trust [24]. Greater trust in strangers is observed in societies where individualism prevails [25]. A meta-analysis showed that interpersonal trust among Chinese college students decreased significantly from 1998 to 2009, possibly due to the uncertainty brought by the rapidly changing social structures and systems in China [26].

However, little is known about how the physical environment affects interpersonal trust. The rapid economic development in many areas around the world, and in China in particular, over the past three decades has brought serious air pollution as reflected in the haze mixed with fog, rife with PM_2.5_ particles. With a diameter of less than 2.5 microns, the particles can easily invade human organs and damage health, particularly the respiratory system [27]. Air pollution has increased the incidence of cardiac dysfunction and cardiovascular diseases [2], the mortality rate of lung cancer patients [28], and abnormal fetal development [29]. It may further have a detrimental effect on interpersonal trust given its negative impact on emotion and other psychological processes that are important for interpersonal trust [10,30].

### 1.3. The Role of Emotion and Emotional Susceptibility

Emotion can change as a function of environmental conditions. A large-scale study conducted with data from social platforms found that temperature, precipitation, temperature differences between day and night, humidity, and cloud cover were all related to emotional expressions on social media sites such as Facebook and Twitter [31]. Specifically, cloudy and rainy weather reduced positive emotions and increased negative emotions, and overheated or overcooled temperatures increased expressions of negative emotions. Another study found that air pollution contributed to annoyance, dissatisfaction, worry, disgust, and other negative feelings [32]. Importantly, emotion plays a critical role in social cognition and behavior [30]. For example, an fMRI study found that participants experiencing aversive moods showed decreased trust in others as well as inhibited activity in the TPJ brain area that is involved in understanding others’ intentions [33]. Taken together, air pollution may intensify negative emotions, which, in turn, reduce interpersonal trust.

Furthermore, individuals with varying personality traits may react differently to air pollution. In particular, emotional susceptibility, namely the tendency of catching the emotions of others [34], often acts as a buffer in interpersonal relationships. Individuals with high emotional susceptibility are more sensitive to the emotions of others, are more likely to empathize with others, and have a greater ability to understand the emotional expressions of others. In marriage, for example, emotional susceptibility is associated with greater marriage satisfaction [35]. Thus, emotional susceptibility may serve as a protective moderator against the negative effect of air pollution on interpersonal trust, such that individuals who are highly emotionally susceptible may be less likely to reduce their trust due to air pollution.

### 1.4. The Present Research

We conducted three studies to examine how air pollution, particularly haze, affected interpersonal trust, and to further examine the roles of emotion and emotional susceptibility as the associated mechanisms and boundary conditions for the effect of air pollution. Study 1 used an experimental manipulation to examine whether viewing pictures of hazy conditions would reduce participants’ levels of interpersonal trust. Study 2 combined data from meteorological monitoring and web crawler technology to analyze messages on an online social platform Weibo and to further establish the link between haze and emotions expressed online. Study 3 simulated Weibo communications to examine how emotion and emotional susceptibility affected the relation between air pollution and interpersonal trust. We expected that air pollution would lead to decreased interpersonal trust (Study 1) as well as decreased positive emotions and increased negative emotions expressed on social media (Study 2), and that the relation between air pollution and interpersonal trust would be mediated by negative emotions and moderated by emotional susceptibility (Study 3).

## 2. Study 1: Haze and Interpersonal Trust

Study 1 aimed to examine the effect of haze on interpersonal trust through an experimental manipulation. We expected that haze would reduce interpersonal trust.

### 2.1. Method

#### 2.1.1. Participants

A total of 110 undergraduate students at Peking University participated (45 men, *M*_age_ = 22.39, *SD*_age_ = 2.58). The planned sample size was 90, based on a power analysis using G*Power (Erdfelder, Bonn, Germany; Faul, Kiel, Germany; Buchner, Trier, Germany) for a between-groups comparison of two groups, power of 0.80, and an effect size of Cohen’s d = 0.60. Participants were from diverse academic disciplines and were recruited through the Bulletin Board System of Peking University. Each participant received 20 CNY for their participation.

#### 2.1.2. Procedure

**Figure 1:**
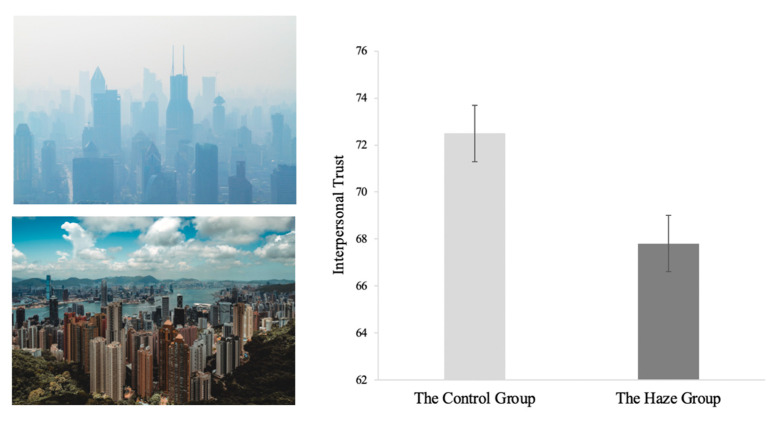
Hazy and clear scenes (left panel) and interpersonal trust scores by condition (right panel). Error bars represent 95% confidence intervals of the means. Haze reduced interpersonal trust. Public domain photos downloaded from https://unsplash.com for illustrative purpose (accessed on 24 May 2021).

A between-subjects design was used. Participants were individually tested in the lab. They accessed and performed the task on their own mobile phones. Participants were randomly assigned to a haze group (*n* = 53; 25 men) or a control group (*n* = 57; 20 men). Both groups first read an article about the dangers of poor air quality, which helped to ensure that all participants had the same background knowledge. Then, the haze group viewed 7 pictures of hazy scenes, and the control group viewed 7 pictures of clear scenes (see illustrative examples in Figure 1 left panel). These pictures were selected from real photos posted by Weibo users. Visibility varied in these pictures, representing different levels of air pollution. Each picture was further accompanied by a text description. For example, a depiction of mild pollution was described as causing “mild aggravation of symptoms in susceptible populations and irritation symptoms in healthy populations.” While viewing the pictures, participants were asked to judge the air quality and pollution depicted in each picture on a 6-point scale (1 = excellent, 2 = good, 3 = mild, 4 = moderate, 5= heavy, 6 = severe), with higher scores indicating more severe air pollutions. Seven, but not more, pictures were used to avoid unnecessary fatigue or boredom among participants.

After rating the pictures, participants completed an Interpersonal Trust Scale (ITS) [12]. The scale consists of 25 items measuring interpersonal trust (e.g., “Parents usually can be relied upon to keep their promises,” and “The absence of teachers during the exam may cause more people to cheat”–reverse scored). Participants rated the items on 5-point scales from 1 (completely disagree) to 5 (completely agree). The scale showed adequate internal consistency, with the Cronbach’s α = 0.76 in the current sample. A score of interpersonal trust was summed across the items, with higher scores indicating greater trust. Data of Study 1 can be found in Supplementary Materials.

### 2.2. Results and Discussion

The haze group (*M* = 4.09, *SD* = 0.69) rated air pollutions in the 7 pictures they viewed as more severe than did the control group (*M* = 2.09, *SD* = 0.40), *F*(1, 108) = 353.19, *p* < 0.001, η_p_^2^ = 0.77. This suggests that the experimental manipulation was effective. Preliminary analyses showed that gender and age had no significant effect on interpersonal trust. Gender and age were therefore not considered further in analysis.

A one-way ANOVA on the interpersonal trust score showed that the haze group had significantly lower scores of interpersonal trust than the control group, *F*(1, 108) = 7.66, *p* = 0.007, η_p_^2^ = 0.07 (see Figure 1 right panel).

Thus, participants who were primed with scenes of air pollution showed lower interpersonal trust than those in the control, which suggests that air pollution reduces interpersonal trust. This is an important addition to previous findings that polluted environments evoke increased indifference to strangers, reduced prosocial behavior, withdrawal from the outside world, increased protection of selfish interests, and augmented aggression [9,36,37,38].

Why does air pollution reduce interpersonal trust? One possibility is that haze generates unpleasant emotions, which then increase the perception of mistrust [39]. Emotion may thus be a mediator between haze and interpersonal trust. To address this question, Study 2 aimed to establish the link between haze and emotion using big data methodology.

## 3. Study 2: Haze and Emotions Expressed in Social Media

In Study 2, we used web crawler technology to obtain data regarding emotions expressed on Weibo. We collected the data in 7 cities with varied degrees of air pollution. We also used meteorological monitoring data to obtain the air quality data of these cities.

### 3.1. Method

#### 3.1.1. Data Collection

Data were collected from January 2 to January 5, 2019, for 4 days. During this time, the northern cities in China suffered from severe haze, whereas the southern cities in China had relatively clear conditions. Seven cities—Beijing, Xi ‘an, Taiyuan, Chongqing, Nanjing, Shanghai, and Guangzhou—were particularly suitable for the study because they have different levels of average air quality in the winter according to historical data: Guangzhou, Shanghai, and Chongqing have generally good air quality; Nanjing and Taiyuan have mild to moderate pollution; whereas Xi ‘an and Beijing experience high levels of air pollution. Weibo is one of the most popular social media sites in China, so its contents are highly representative. We collected data on Weibo at the end of each day, exporting all posts during that day that were related to the 7 cities (see detail below). We obtained a total of 1036 Weibo posts from the 7 cities during the 4-day period. At the same time, air quality data of each city on each of the 4 days were obtained through the World Air Quality Website (http://aqicn.org; accessed on 5 January 2019), including real-time AQI (air quality index), minimum AQI in the past 48 h, and maximum AQI in the past 48 h. The greater AQI value indicates the higher level of air pollution.

#### 3.1.2. Data Coding

Because our focus was on people’s emotions, we excluded public and celebrity accounts as well as Weibo contents with multiple city tags or unknown posting locations. This resulted in 644 Weibo posts for coding and analysis. Trained research assistants coded the posts for positive (e.g., happiness, joy, peacefulness) and negative emotions (e.g., sadness, anger, fear, disgust, disappointment) [40]. Based on the emotional terms and emojis in a Weibo post, a coder tallied the frequencies of positive and negative emotions, respectively. Another two independent coders randomly checked the coded data and confirmed the coding accuracy. Each Weibo post received a score of positive emotion and a score of negative emotion. The Weibo emotion scores were matched with the air quality data of the same day. Data of Study 2 can be found in Supplementary Materials.

### 3.2. Results and Discussion

The air pollution statistics in the 7 cities averaged across the 4 days are presented in Table 1. First, we examined how air quality was related to emotions expressed on Weibo. Across the 4 days, 298 (46.3%) posts contained words indicating positive emotions (range = 0–12), while 95 (14.8%) contained words indicating negative emotions (range = 0–5). Descriptive data and correlations between online emotion expressions and real-time AQI (air quality index), minimum AQI in the past 48 h, and maximum AQI in the past 48 h are reported in Table 2. Positive emotions were negatively correlated with real-time AQI and the maximum AQI in the past 48 h, while negative emotions were not significantly correlated with any AQI indexes. In other words, people expressed fewer positive emotions online when air pollution was worse.

We conducted additional analyses to examine the impact of air pollution on emotions. According to the World Air Quality Website, air pollution levels varied from excellent, good, mild, to moderate, heavy, and severe. We classified excellent, good, and mild levels as non-obvious pollution, and moderate, heavy, and severe levels as obvious pollution. Under the obvious pollution condition, 40% of the emotions expressed on Weibo were positive, whereas under the non-obvious pollution condition, 54% of the emotions were positive, χ^2^(1) = 13.27, *p* < 0.001. Thus, Weibo users were less likely to express positive emotions under polluted than non-polluted conditions. The expression of negative emotions did not differ significantly between the two conditions.

Taken together, results of Study 2 showed that the expression of positive emotions was correlated with air quality and that haze reduced expression of positive emotions. Interestingly, haze and air pollution did not increase the expression of negative emotions online. It is possible that people are conscientious about being positive on social media, given the online social norms of adherence to positive emotions [41]. It has been shown that Facebook users express more positive than negative emotions [42] and present themselves as happier than they actually are [43]. Accordingly, people might have experienced increased negative emotions associated with haze but did not express the emotions online.

We have shown that haze reduced interpersonal trust (Study 1) and the expression of positive emotions on social media (Study 2). The next question is what role emotion plays in the relation between haze and interpersonal trust. Furthermore, although people have easy access through social media to information about air pollution, they may have diverse reactions to the information as a result of their emotional susceptibility. Using the social media context and an experimental method, we examined in Study 3 whether the relation between haze and interpersonal trust was mediated by emotion and moderated by emotional susceptibility.

## 4. Study 3: The Roles of Emotion and Emotional Susceptibility

We simulated the Weibo environment in the laboratory in Study 3. To understand the mechanisms underlying the relation between haze and interpersonal trust, we experimentally manipulated perceptions of haze and examined the roles of emotion and emotional susceptibility.

### 4.1. Method

#### 4.1.1. Participants

Participants were 91 undergraduates from Peking University (24 men; *M*_age_ = 23.98, *SD*_age_ = 4.46). The planned sample size was 90, based on a power analysis using G*Power (Erdfelder, Bonn, Germany; Faul, Kiel, Germany; Buchner, Trier, Germany) for a between groups comparison of two groups, power of 0.80, and an effect size of Cohen’s d = 0.60. Participants were from diverse academic disciplines and were recruited through the Bulletin Board System of Peking University. They each received 20 RMB for their participation.

#### 4.1.2. Procedure

A between-subjects design was used. Participants were individually tested in the lab. They accessed and performed the task on their own mobile phones. They were randomly assigned to a pollution group (*n* = 45; 13 men) or a non-pollution group (*n* = 46; 11 men). Participants were asked to imagine that they were browsing Weibo on the topic “talk about your day” using their mobile phones. They were presented with 12 Weibo messages posted by others and were asked to browse these messages. The messages were selected to assimilate a realistic Weibo context, with 6 of them containing various emojis and symbols. They were on average 57 words in length. A pilot test showed that participants could process 12 messages at a time without distraction. Participants were instructed to rate the emotional intensity of each message on a 6-point scale from 1 (not strong at all) to 6 (very strong). Participants in the pollution group read 7 messages describing the daily life and 5 messages discussing air pollutions. Participants in the non-pollution group read the same 7 messages describing the daily life and 5 messages describing other topics unrelated to air pollutions. After the viewing, participants were asked to imagine themselves participate in a discussion on this topic (i.e., “talk about your day”), and to write a post on Weibo.

Participants then completed the 15-item Emotional Contagion Scale (ECS) that measures emotional susceptibility to love, happiness, fear, anger, and sadness (e.g., “When the person I’m talking to cry in front of me, I can’t help crying,” and “If someone around is angry, it will make me feel unhappy”) [34]. They rated the items on 5-point scales from 1 (never) to 5 (always). Cronbach’s α = 0.79 in the current sample. The scores were summed across the items to index emotional susceptibility. Participants also completed the Positive and Negative Affect Scale (PANAS) [44]. It includes positive affect (PA) and negative affect (NA) subscales, each containing 10 emotional adjectives. Participants rated each emotion on a 5-point scale according to the degree of emotional intensity they experienced at that moment (1 = almost nothing, 5 = extremely strong). Cronbach’s α = 0.90 for PA, and Cronbach’s α = 0.89 for NA. Each participant received a PA score and a NA score. Finally, participants completed the Interpersonal Trust Scale (ITS) same as in Study 1, with Cronbach’s α = 0.69 [12]. Data of Study 3 can be found in Supplementary Materials. 

### 4.2. Results and Discussion

For manipulation check, each participant’s ratings on the emotional intensity of the 12 Weibo messages were summed. Participants in the pollution group (*M* = 44.87, *SD* = 6.63) rated significantly higher levels of emotional intensity than those in the non-pollution group (*M* = 39.50, *SD* = 6.25), *F*(1, 89) = 15.79, *p* < 0.001, η_p_² = 0.15. The manipulation was thus effective.

Preliminary analyses showed that women scored higher than men on emotional susceptibility, *t*(89) = 2.12, *p* = 0.036, *d* = 0.50, and age was significantly correlated with PA, *r* = 0.23, *p* = 0.03. Gender and age were therefore included as covariates in relevant analyses. Notably, NA was negatively correlated with interpersonal trust, while PA was positively correlated with emotional susceptibility (see Table 3). In other words, participants who reported more negative affect exhibited less interpersonal trust and those who reported more positive affect scored higher on emotional susceptibility.

**Figure 2:**
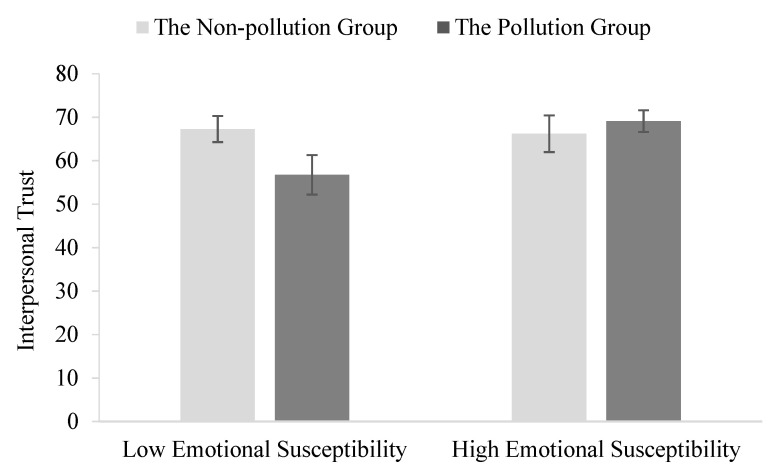
The interaction between air pollution and emotional susceptibility on interpersonal trust. Error bars represent 95% confidence intervals of the means. Participants who scored low on emotional susceptibility showed reduced interpersonal trust in the air pollution condition.

To examine whether emotional susceptibility moderated the effect of air pollution on interpersonal trust, we divided standardized emotional susceptibility scores into high and low groups according to the mean plus/minus 1 standard deviation. Whereas air pollution negatively predicted interpersonal trust in the low emotional susceptibility group, *B* = −0.74, *SE* = 0.30, *p* = 0.01, it had no significant effect on interpersonal trust for the high-susceptibility group (see Figure 2). Thus, emotional susceptibility moderated the negative effect of air pollution on interpersonal trust such that high emotional susceptibility seemed to serve as a protective factor.

Next, we tested the mediation role of emotion for the effect of air pollution on interpersonal trust. The pollution group (*M* = 28.58, *SD* = 6.92) and the non-pollution group (*M* = 27.78, *SD* = 6.81) did not differ significantly in PA, *t*(89) = −0.55, *p* = 0.58, *d* = −0.12. However, the pollution group (*M* = 21.11, *SD* = 8.49) scored significantly higher on NA than the non-pollution group (*M* = 17.37, *SD* = 6.0), *t*(89) = −2.42, *p* = 0.02, *d* = −0.51, which suggests that air pollution evoked negative emotions in the pollution group. NA was thus included in the mediation analysis.

We examined an overall research model using Hayes’s [45] model 5 in the Process of SPSS for the overall model bootstrap test, with gender as a covariate, pollution condition as the independent variable, interpersonal trust as the dependent variable, NA as the mediator, and emotional susceptibility as a moderator.

**Figure 3:**
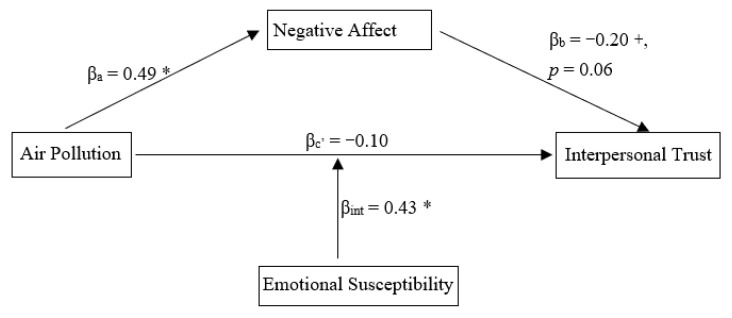
The overall research model. The relation between air pollution and interpersonal trust was mediated by negative affect and moderated by emotional susceptibility. ^+^ *p* = 0.06, * *p* < 0.05.

The overall model test showed that air pollution significantly predicted NA. NA had a marginally significant effect on interpersonal trust. The effect of pollution condition on interpersonal trust was reduced to being non-significant once NA was included in the model. Air pollution condition interacted with emotional susceptibility to have a significant predictive effect on interpersonal trust (see Figure 3). Both the mediating effect of negative emotion and the moderating effect of emotional susceptibility were confirmed.

Thus, air pollution weakened interpersonal trust by increasing negative emotions, which then reduced interpersonal trust. Furthermore, emotional susceptibility moderated the impact of air pollution on interpersonal trust. Individuals high on emotional susceptibility are generally better able to understand others and are less influenced by external environments when making judgments [35]. Accordingly, air pollution did not reduce their interpersonal trust. In contrast, individuals low on emotional susceptibility were more easily influenced by air pollution to breed distrust.

## 5. General Discussion

In three studies, we found empirical support for our hypotheses that haze reduces interpersonal trust, negative emotion mediates the haze effect on interpersonal trust, and emotional susceptibility moderates the haze effect on interpersonal trust. These original findings add critical evidence for the negative impact of air pollution on psychological outcomes and have important real-life implications.

In Study 1, we experimentally manipulated perceptions of air pollution and found lower interpersonal trust among participants in air pollution conditions. This finding extends extant research showing that air pollution evokes indifference and hostility towards others [36,37,38].

In Study 2, we used big data methodology to further examine the influence of haze on emotions. By analyzing data of air quality from the World Air Quality Website and of emotions expressed in Weibo posts from 7 cities of varied degrees of pollution, we found that haze was associated with reduced expressions of positive emotions, although haze did not increase expressions of negative emotions. The latter result may reflect people’s tendency to express positive rather than negative emotions online in an effort to conform to social norms [41]. The relation of haze conditions to reduced positive emotions in everyday life is important as it may have long-term consequences for mental health and psychosocial adjustment [11,46].

Study 3 further examined the roles of emotion and emotional susceptibility in the relation between air pollution and interpersonal trust. As expected, air pollution reduced interpersonal trust through the mediating effect of negative emotions. When haze causes negative emotions, people are likely to focus on others’ negative characteristics and thus show reduced interpersonal trust [39]. Furthermore, emotional susceptibility moderated the relationship between air pollution and interpersonal trust. People who have higher levels of emotional susceptibility often interpret others’ emotions and motivations in a positive light, which can then facilitate positive interpersonal interactions [34,35]. Emotional susceptibility appears to serve as a protective buffer against the negative effect of air pollution on interpersonal trust. In contrast, people with low emotional susceptibility tend to be insensitive to others, which may make them more vulnerable to the influence of external factors. Thus, adverse environmental factors like air pollution are more likely to decrease their trust in others.

### 5.1. Implications

This research sheds new light on how environmental factors affect human psychological processes. Utilizing experimental and big data methods, the studies provide critical evidence for the impact of air pollution on interpersonal trust. The finding that emotion serves as a mediator that gives rise to the effect of air pollution on interpersonal trust further highlights the important role of emotion in social cognition and behavior [30,39,47]. The finding of emotional susceptibility as a moderator for the effect of air pollution on interpersonal trust suggests important individual differences that can inform targeted interventions. Furthermore, the findings have implications for everyday practices. When we interact with others in an air-polluted environment, we need to be more sensitive to each other’s emotional experiences, increase effective communication, maintain positive feelings, and together build and facilitate trust [48]. From a societal perspective, the reduction of interpersonal trust caused by air pollution can have detrimental effects on social cohesion and harmony. Improving air quality is thus not only important for individuals but also beneficial for society as a whole. Policymakers should develop both short-term and long-term plans to address air pollution problems, increase open communication with the public, and actively facilitate interpersonal trust and social harmony in the joint effort against air pollution.

### 5.2. Limitations

There are important limitations in the current studies that need to be addressed in future research. In particular, social media users often selectively express emotions and do not always accurately report their real emotional experiences online. Additional research is needed to examine emotions in reaction to air pollution both online and offline. Furthermore, air pollution is known to have long-term effects on human conditions [6,7,11]. Future studies should thus examine long-term consequences of haze and other forms of air pollution for interpersonal trust and other psychosocial outcomes. Moreover, Study 2 data collection took place on January 2 through January 5, right after the New Year’s Day holiday. As a result, people might experience the Monday blues phenomenon. This experience, however, would apply to people living in both polluted and unpolluted areas. It was therefore not a confound to air quality and should not affect our results. Nevertheless, it will be interesting to examine in future research the effect of air pollution in interaction with other ecological factors, such as the days of the week, on psychological experiences. In addition, Studies 1 and 3 used college samples, which may limit the generalizability of the findings. Future studies should include more diverse populations to better understand the impact of air pollution on mental states and behavior, and also examine individual differences (e.g., personality traits, the presence of significant anxiety-depression, drug use) that can affect the vulnerability to the negative impact of air pollution.

## 6. Conclusions

The present research combines experimental and big data methods and provides new insights into how the external environment affects human psychosocial functioning. Air pollution in the form of haze reduced interpersonal trust (Study 1) and positive emotions expressed on social media (Study 2). Negative emotion played a mediating role and emotional susceptibility played a moderating role for the effect of air pollution on interpersonal trust (Study 3). Together, the findings raise awareness of the harmful psychological and interpersonal impacts of air pollution and of the importance of improving air quality for enhancing interpersonal trust and social harmony.

## Figures and Tables

**Table 1:** Air pollution statistics in the 7 cities.

City	AQI	Air Pollution Level	The Minimum AQI in the Past 48 h	The Maximum AQI in the Past 48 h
Guangzhou	114	mild level	82	138
Shanghai	121	mild level	55	159
Nanjing	163	moderately polluted	73	217
Chongqing	154	moderately polluted	59	162
Xi‘an	293	heavily polluted	139	354
Beijing	109	mild level	27	228
Taiyuan	193	moderately polluted	113	294

Note: AQI refers to Air Quality Index.

**Table 2:** Descriptive data and correlations between Weibo emotions and air quality data.

	*M*	*SD*	1	2	3	4	5
1 Positive emotion	0.71	1.04	—				
2 Negative emotion	0.25	0.68	−0.07	—			
3 Real-time AQI	160.19	70.47	−0.16 **	0.06	—		
4 The minimum AQI in the past 48 h	72.63	37.48	−0.02	0.04	0.42 **	—	
5 The maximum AQI in the past 48 h	204.29	81.26	−0.12 **	0.04	0.68 **	0.50 **	—

Note. * *p* < 0.05, ** *p* < 0.01, *** *p* < 0.001.

**Table 3:** Descriptive data and correlations of the variables.

	*M*	*SD*	1	2	3
1 Negative affect	19.22	7.56			
2 Positive affect	28.18	6.84	−0.02		
3 Emotional susceptibility	55.22	7.56	0.09	0.32 **	
4 Interpersonal trust	67.81	8.54	−0.22 *	0.07	0.16

Note. * *p* < 0.05, ** *p* < 0.01.

## Data Availability

The data presented in this study are available in Supplementary Materials.

## Supplementary Materials

The following are available online at https://www.mdpi.com/article/10.3390/ijerph18115631/s1, Data S1: Study 1 data, Data S2: Study 2 data, Data S3: Study 3 data.
